# A national intervention to reduce imaging for low back pain by general practitioners: a retrospective economic program evaluation using Medicare Benefits Schedule data

**DOI:** 10.1186/s12913-019-4773-y

**Published:** 2019-12-21

**Authors:** Tessa Morgan, Jianyun Wu, Ludmila Ovchinikova, Robyn Lindner, Suzanne Blogg, Rachael Moorin

**Affiliations:** 1 NPS MedicineWise, Sydney, New South Wales Australia; 2 Curtin University, Perth, Western Australia Australia

**Keywords:** Low back pain, Computed tomography (CT), Audit and feedback, Low value care, Economic evaluation, General practitioner education, Choosing wisely

## Abstract

**Background:**

The overuse of diagnostic imaging for low back pain (LBP) in Australia results in unnecessary cost to the health system and, for patients, avoidable exposure to radiation. The 2013 NPS MedicineWise LBP program aimed to reduce unnecessary diagnostic imaging for non-specific acute LBP in the Australian primary care setting. The LBP program delivered referral pattern feedback, a decision support tool and patient information to 19,997 (60%) of registered Australian general practitioners (GPs). This study describes the findings from evaluation of the effectiveness of the 2013 LBP program at reducing X-ray and computed tomography (CT) scans of the lower back, and the financial costs and benefits of the program to the government funder.

**Methods:**

The effectiveness of the 2013 LBP program was evaluated using population-based time-series analysis of administrative claims data of Medicare Benefits Schedule (MBS) funded X-ray and CT scan services of the lower back. The CT scan referral trend of non-GP health professionals was used as an observational control group in a Bayesian structural time-series model. A retrospective cost–benefit analysis and cost-effectiveness analysis was conducted using program costs from organisational records and reimbursement data from the MBS.

**Results:**

The 2013 NPS MedicineWise LBP program was associated with a statistically significant 10.85% relative reduction in the volume of CT scans of the lumbosacral region, equating to a cost reduction to the MBS of AUD$11,600,898. The best available estimate of program costs was AUD$141,154. Every dollar of funding spent on the 2013 LBP program saved AUD$82 of funding to the MBS for CT scan reimbursements. Therefore, from the perspective of the Australian Government Department of Health, the 2013 LBP program was cost saving. The program cost AUD$2.82 per CT scan averted in comparison to the scenario of no program. No association between the 2013 NPS MedicineWise LBP program and the volume of X-ray items on the MBS was observed.

**Conclusions:**

The 2013 NPS MedicineWise LBP program reduced CT scan referral by GPs, in line with the program’s messages and clinical guidelines. Reducing this low-value care produced savings to the health system that exceeded the costs of program implementation**.**

## Background

Low back pain (LBP) is a highly prevalent health problem. The 1-year prevalence of LBP is estimated to be 38% worldwide [1] and 68.9% in Australia. [2] Low back pain refers to pain and discomfort affecting the lumbar and/or sacral regions of the back. Based on the duration of the episode, LBP can be classified as acute (less than 6 weeks), subacute (6–12 weeks) or chronic (more than 12 weeks).

In primary care in Australia, general practitioners (GPs) act as gatekeepers to various treatment pathways. Patients require GP referral to access care from specialist physicians and to receive government-subsidised imaging services outside of the hospital setting. In 2015–16, management of back complaints accounted for 3.1% of Australian general practice encounters. [3] While 95 to 99% of patients who present to GPs with LBP do not have a serious underlying spinal pathology, [4] the investigation and treatment of these patients significantly impacts health expenditure. The potential for rare but severe conditions associated with acute LBP, such as spinal fracture and cancer, means that accurate diagnosis is important.

Alerting features (‘red flags’) for serious conditions associated with acute LBP have been published in Australian guidelines. [5] There is no evidence that imaging in the absence of red flags, referred to as non-specific LBP, improves patient outcomes or alters clinical decision making. [5] For more than a decade, Australian guidelines have advised against diagnostic imaging for routine assessment of patients with non-specific LBP. [5] Despite this, a 2010 Australian study found that over one quarter of patients presenting to their GP with a new episode of LBP were referred for diagnostic imaging. [6] In 2009–2012 a national survey of GP consultations reported referral for diagnostic imaging at 16.8% of all consultation that dealt with a back complaint, including new and ongoing. The rate was similar to that recorded in 2002-2005, however between these time points the rate of CT scans for back complaints significantly increased from 4.7 to 6.1 CT referrals per 100 of all consultation that dealt with a back complaint [7]. The use of diagnostic imaging for LBP outside of guideline recommendations results in unnecessary cost to the Australian health system and, for patients, avoidable unnecessary exposure to radiation.

In Australia, diagnostic imaging in the community is subsidised by the Australian Government via the Medicare Benefits Scheme (MBS). General practitioners can refer patients for MBS-subsidised computed tomography (CT) scans and X-rays, but not MBS-subsidised Magnetic Resonance Imaging (MRI) of the lower back. However, specialist physicians can refer patients for MBS-subsidised MRI of the back, or GPs can refer patients if they agree to pay the entire cost. A combination of referrer restrictions, specific clinical requirements for MRIs scans which can be MBS-subsidised and a licensing system unique to MRI machines contributes to a lower volume of MRI use compared to CT and x-ray modalities. [8] The use of CT scans for imaging of the lumbar spine region has increased substantially since being listed on the Medicare Benefits Schedule in 2001. Between 2002 and 2010, it rose from 886 to 1131 items per 100,000 population and the cost to the Australian Government rose from approximately AUD$35 million to AUD$56 million. [9] While some increased use is due to Australia’s aging population, change in practice has also contributed. [10]

The use of CT scans represents significant diagnostic value but also potential risk. A positive association between exposure to CT scans and cancer risk has been reported in cohort studies of British [11] and Australian [12] children and adolescents. The Australian study used linked data from a cohort of 10.9 million Australian children and adolescents with a mean follow-up 9.5 years after exposure to a CT scan. [12] After accounting for age, sex and year of birth, the overall cancer incidence was 24% higher in those exposed to CT scans compared with those who were not exposed. In the population exposed to CT scans there was an excess of 608 cancers and an absolute excess incidence rate of 9.38 per 100,000 person-years at risk. The causal relationship between CT scans and cancers was further supported by an observed positive correlation between the site of the CT scan and the site of the cancer. [12] The dose–response relationship quantified in the study, was similar to the quantification reported in the National Research Council’s BEIR VII Phase 2 report, using the linear-no-threshold model theory of health risk from radiation exposure. [13]

The inappropriate use of imaging for non-specific LBP is an important topic in the discussion about low value care in Australia. Through the Choosing Wisely Australia initiative, The Royal Australian and New Zealand College of Radiologists and the Australian Physiotherapy Association both include a recommendation to not request or perform imaging for patients with non-specific LBP with no indication of a serious cause. [14, 15] Different forms and intensities of interventions to change the practice of Australian GPs have previously been evaluated, including the mailed dissemination of guidelines [16] and interactive workshops. [17] Systematic reviews point toward a lack of clear direction on the most effective strategy to address the inappropriate use of diagnostic imaging in people with LBP and the need for better evaluation of the different interventions. [18–20]

NPS MedicineWise has been promoting the quality use of medicines in Australia since 1998. NPS MedicineWise is an independent organisation whose programs are funded by the Australian Government Department of Health. Since 2009, NPS MedicineWise has also sought to provide accurate, balanced, evidence-based information and services to health professionals and the community on the quality use of diagnostic tests. [21]

In 2013, NPS MedicineWise implemented the LBP program. The objective of the program was to educate consumers and GPs about the appropriate management and use of imaging tests for acute LBP. It built on previous NPS MedicineWise programs with the same objective, including one implemented in 2010. The anticipated outcome of the 2013 NPS MedicineWise LBP program was a reduction in unnecessary GP referrals for CT scans and X-rays of the lower back.

In this study, we conducted an economic evaluation of the 2013 NPS MedicineWise LBP program. We aimed to identify the financial costs and benefits of the program to the Australian Government Department of Health and the program’s cost-effectiveness at reducing GP referrals for diagnostic imaging of the lower back.

## Methods

The program effectiveness study was a retrospective population-based cohort study using time-series analysis based on routinely collected practitioner level MBS data for the period May 2010 to February 2015, inclusive. The results informed the retrospective cost-effectiveness study. The reporting of this study is based on the REporting of studies Conducted using Observational Routinely-collected health Data (RECORD) statement. [22]

### NPS MedicineWise low Back pain program

The program was an educational feedback intervention which aimed to reduce inappropriate CT scans and X-rays of the lower back by improving GPs’ referral practice for acute LBP presentations. Consistent with Australian clinical guidelines, [5] the key message of the program was that diagnostic imaging tests should not be used in the routine assessment of patients with acute LBP in the absence of red flags. The program comprised of a personalised MBS feedback report, a symptom self-management prescription pad, and an online decision support tool.

In June 2013, the personalised MBS feedback report was sent to 19,997 registered practising GPs in Australia, 60% of the 33,337 Australian GPs. [23] The feedback report was populated with data from the Australian Government Department of Human Services (DHS). Selection of GPs was based on a threshold of reimbursement, to indicate active use of the targeted MBS items. The report compared an individual GP’s referral practices, for MBS items for CT scan and X-ray of the low back, with an average for other GPs in a similar geographical area, based on the Rural, Remote and Metropolitan Area classification. Feedback data was accompanied by educational messages and points for reflection based on clinical guidelines and current relevant evidence.

The cover letter of the feedback report promoted free resources available from NPS MedicineWise to support GPs in managing LBP consultations, including managing patient expectations. The symptom self-management prescription pad was a pad of tear-off patient information sheets on how to manage acute LBP, including information about exercise, pain relief and imaging tests. The patient information sheets had space for the GP to write personalised advice.

The *Back Pain Choices* online decision support tool, based on a four step decision algorithm, assisted GPs in the diagnosis and management of LBP. A personalised information sheet for the patient could be printed out. The tool was developed in collaboration with the George Institute for Global Health and published online in 2012. [24]

### Imaging utilisation analysis

#### Imaging utilisation data

This study used the MBS national administrative database. The Australian government subsidises medical services that are listed on the MBS via Australia’s universal healthcare system, the Medicare Scheme. Listed imaging tests that occur outside of the public hospital setting are subsided by Medicare if an appropriate medical provider refers the patient. Provider level MBS data for CT scanning of the lumbosacral region (MBS item number: 56223) and X-ray of the spine lumbosacral and sacrococcygeal region (MBS item numbers: 58106 and 58109) were requested and provided by the DHS. The data contained the number of services and amount of benefit paid for each MBS item number grouped by individual referring provider and by month of service from May 2010 to February 2015. Since Australian GPs and GP registrars were the target population for the program and the cohort for this study, we requested that each referring provider in the data be classified by the DHS as either a GP or non-GP (e.g. specialist physicians) according to a provider’s listed major specialty.

#### Outcomes measures

The study evaluated the effectiveness of the NPS MedicineWise program at reducing the number of CT scans and X-rays of the low back referred by GPs. The MBS data was summarised as the total number of services and total amount of benefit paid each month for GPs and non-GPs. The total monthly volumes were used to measure the program’s effectiveness at changing utilisation and the total monthly amount of benefit reimbursed was used to analyse the monetary benefit associated with the program’s effect.

#### Statistical analysis

A Bayesian structural time-series model (BSTM) [25, 26] was fitted to describe and quantify the association between the NPS MedicineWise LBP Program and the changes in monthly services and benefit paid for CT scans and X-rays referred by GPs. Data between May 2010 and May 2013 were modelled, with data from non-GPs used as a predictor, and the temporal trends from June 2013 to February 2015 were forecasted. The estimated change in the number of services and benefits paid following the intervention were calculated from the monthly differences between observed and forecasted results. Time-series components of the BSTM incorporated trend and seasonality with a structural model containing a regression component and static coefficient. A detailed discussion of prior specification and elicitation for unknown parameters in the model has been previously published. [26] The application of this method to Australian administrative health databases has been previously published. [27]

The BSTM method generalised the difference-in-difference approach to time-series analysis by forecasting the post-intervention period with a linear control predictor. [26] The underlying assumption, as with the difference-in-difference method, is that the ‘observational control group’ is an adequate proxy for the level observed without the intervention. [26] This relies on the ‘parallel trend’ assumption, that in the absence of treatment, the treatment group and the comparison group would follow parallel paths over time. [28] This assumption allows difference-in-difference to account for unobserved variables, which are assumed to remain fixed over time. [29] The validity of including monthly service volume and benefit payment from non-GPs in forecasting those for GPs was dependent on the following assumptions. First, from the intervention perspective, the referring behaviour of non-GPs was less likely to be influenced by the intervention. An assumption based on the fact that non-GPs did not receive the intervention. Second, from the observed data, a stationary relationship (similar trend and seasonal pattern) in data between GPs and non-GPs could be established prior to the interventions. Stationarity prior to the intervention was the primary assumption of our modelling approach. [26] The time-series analyses were conducted using the “CausalImpact” package [25] in the statistical software R [30].

### Economic evaluation

#### Program cost data

Program development and implementation costs were retrospectively calculated from NPS MedicineWise organisational records. Financial records were used to capture invoiced costs such as production, data, printing and distribution of the individualised MBS feedback intervention, the production and printing of the symptom self-management prescription pad, and out sourced development costs associated with the online decision aid tool. NPS MedicineWise staff costs were estimated from project management forecasting documents, timesheet data for equivalent tasks and staff salary data.

Since similar products are regularly developed and implemented at NPS MedicineWise, resource data for five similar products were collected to estimate variation associated with the base case estimates. In the variation data based on actuals, the maximum value was 40% higher than the minimum value. For intervention components, which did not have data on similar products, a conservative estimate was applied of 50% higher or lower.

A 25% increase was added to all human recourse costs to account for infrastructure and support services. All costs were adjusted to 2015 Australian dollars using the Consumer Price Index (CPI) published by the Australian Bureau of Statistics (ABS). [31]

#### Economic analysis

The timeframe for the economic evaluation of the program was July 2013 to February 2015, 20 months post-program implementation. The effect of the program on GPs’ CT scan referral was discounted at an annual rate of 5%, calculated monthly after the first year.

The Australian Government Department of Health funds both the MBS and the implementation of NPS MedicineWise quality use of diagnostics educational programs. From the perspective of this funder, the net benefit is the difference between the cost of NPS MedicineWise developing and delivering the program and the change in MBS expenditure on CT scans associated with the program. The monetary costs and benefits of the program from the perspective of the funder, the Australian Government Department of Health, were compared using the net benefit and cost-benefit ratio. The net benefit was calculated by subtracting the adjusted cost of implementing the program from the adjusted and discounted benefit of the program, defined as the change in reimbursement costs to the MBS associated with the intervention. The impact of the program on MBS services utilisation and expenditure was discounted at an annual rate of 5% after year one, calculated monthly. Incremental cost-effectiveness was estimated and compared to a hypothetical scenario of no program implementation.

Sensitivity analysis for the estimates of cost-benefit ratio and incremental cost-effectiveness ratio (ICER) was conducted using estimates of variation in cost, benefit and outcomes derived from the 95% Bayesian credible intervals of MBS time-series analysis and the comparison of program costs to other similar programs delivered by NPS MedicineWise. Sensitivity analysis for the cost-benefit was estimated from the net benefit and cost-benefit ratio for the “best and worst” scenarios using the estimated maximum and minimum cost and benefit.

For the cost-effectiveness analysis, univariate and probabilistic sensitivity analyses were conducted using TreeAge [32]. Univariate sensitivity analysis of the ICER estimates varied the program costs and effect estimates. A probabilistic sensitivity analysis (50,000 iterations) varied the same estimates. Uncertainty around the program costs was included using a gamma distribution. The uncertainty around the effectiveness of the program was estimated using a normal distribution and confidence intervals from the time-series analysis. A willingness to pay threshold of AUD $231 was used to represent the average savings to the MBS from each CT scan averted.

## Results

### Trend in CT scan and X-ray utilisation and reimbursement

**Fig. 1:**
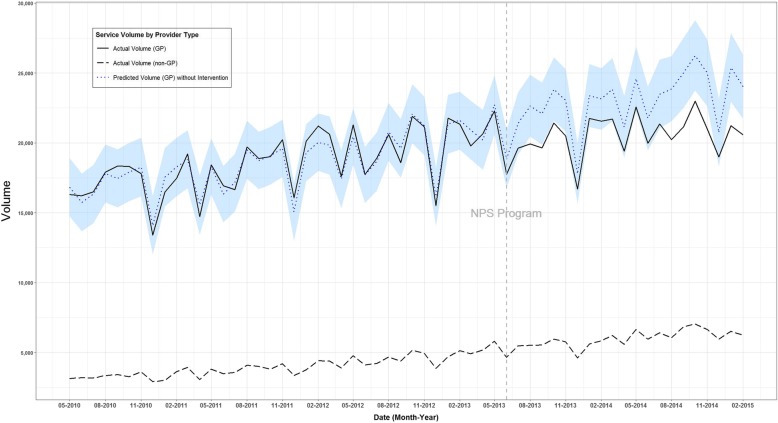
Time series analysis of monthly count of CT-scan service volume (item number 56223), May 2010–February 2015, for general practitioner providers’ actual volume, other health professional providers’ actual volume and general practitioner providers’ predicted volume without intervention

**Fig. 2:**
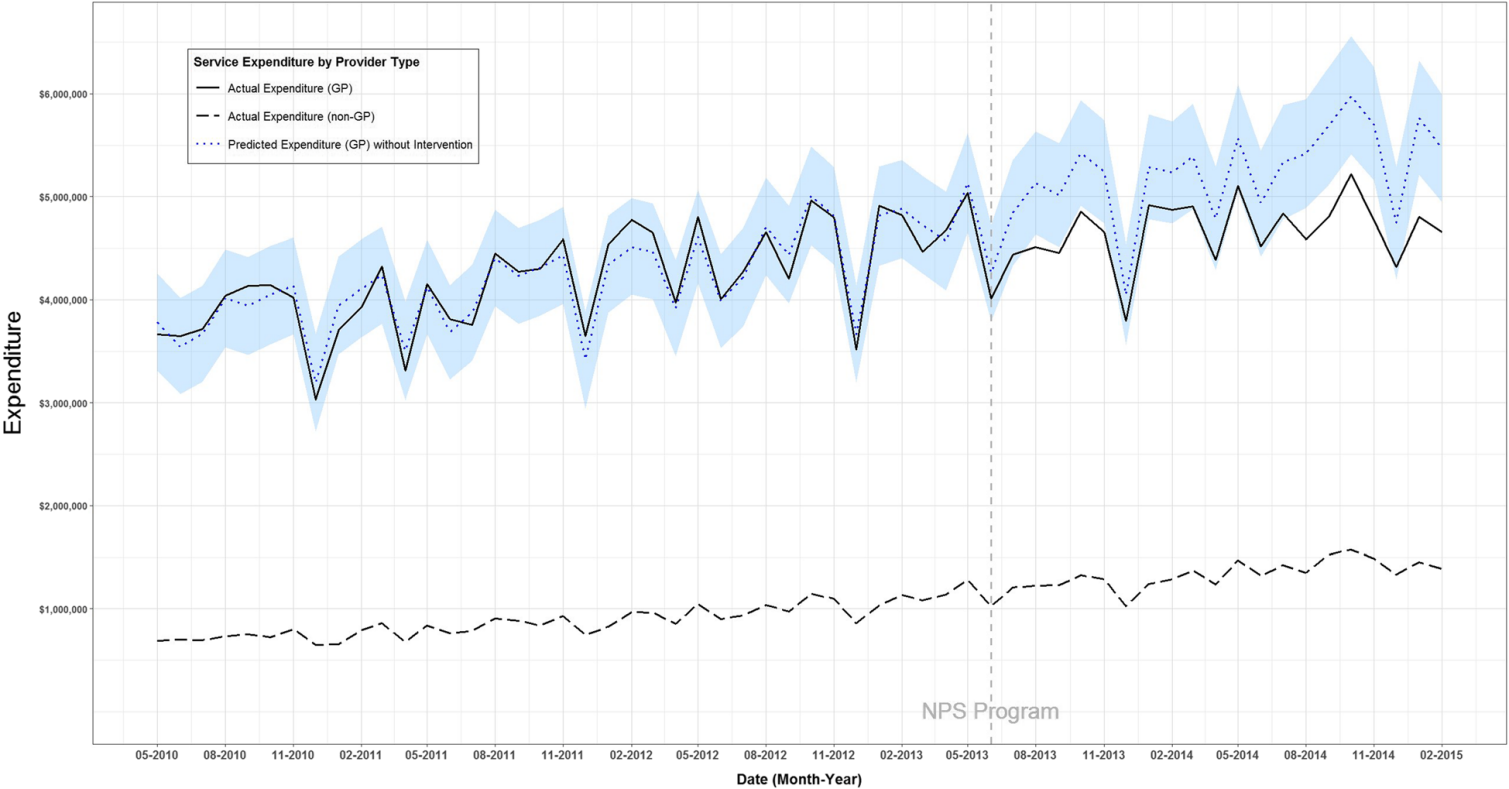
Time series analysis of monthly count of CT-scan service reimbursement (item number 56223), May 2010–February 2015, for general practitioner providers’ actual expenditure, other health professional providers’ actual expenditure and general practitioner providers’ predicted expenditure without intervention

Between May 2010 and February 2015 (57 months), 1,119,796 CT scans of the lumbosacral region (MBS item number 56223) were referred by GPs, with a reimbursement cost of AUD$253 million. There was an upward trend in CT scan service volume (Figure. 1) and reimbursement (Figure. 2) prior to the 2013 NPS MedicineWise LBP program.

The monthly volume of CT scans of the lumbosacral region by referrer (GP or non-GP] and the time point of the NPS MedicineWise intervention are shown in Figure. 1. The modelled counterfactual estimate of CT scan volume without the 2013 NPS MedicineWise LBP program is shown in Figure. 1.

The main intervention of the 2013 NPS MedicineWise LBP program, the MBS feedback report, was mailed to GPs in June 2013. There were 37 monthly timepoints before the intervention and 20 after the intervention included in the analysis period. Following the intervention, the observed volume of CT scans of the lumbosacral region referred by GPs was 10.85% lower than the volume without the intervention estimated by the counterfactual. This reduction was statistically significant. The intervention was associated with an estimated 50,186 fewer GP-referred CT scans between July 2013 and February 2015 (95% posterior interval 3919 to 96,476).

Following the intervention, the observed reimbursement of CT scans of the lumbosacral region referred by GPs was 11.05% lower than reimbursement without the intervention estimated by the counterfactual. This reduction was statistically significant. The intervention was associated with an estimated AUD$11,600,898 cost reduction between July 2013 and February 2015 (95% posterior interval $1,093,011 to $22,100,313).

**Fig. 3:**
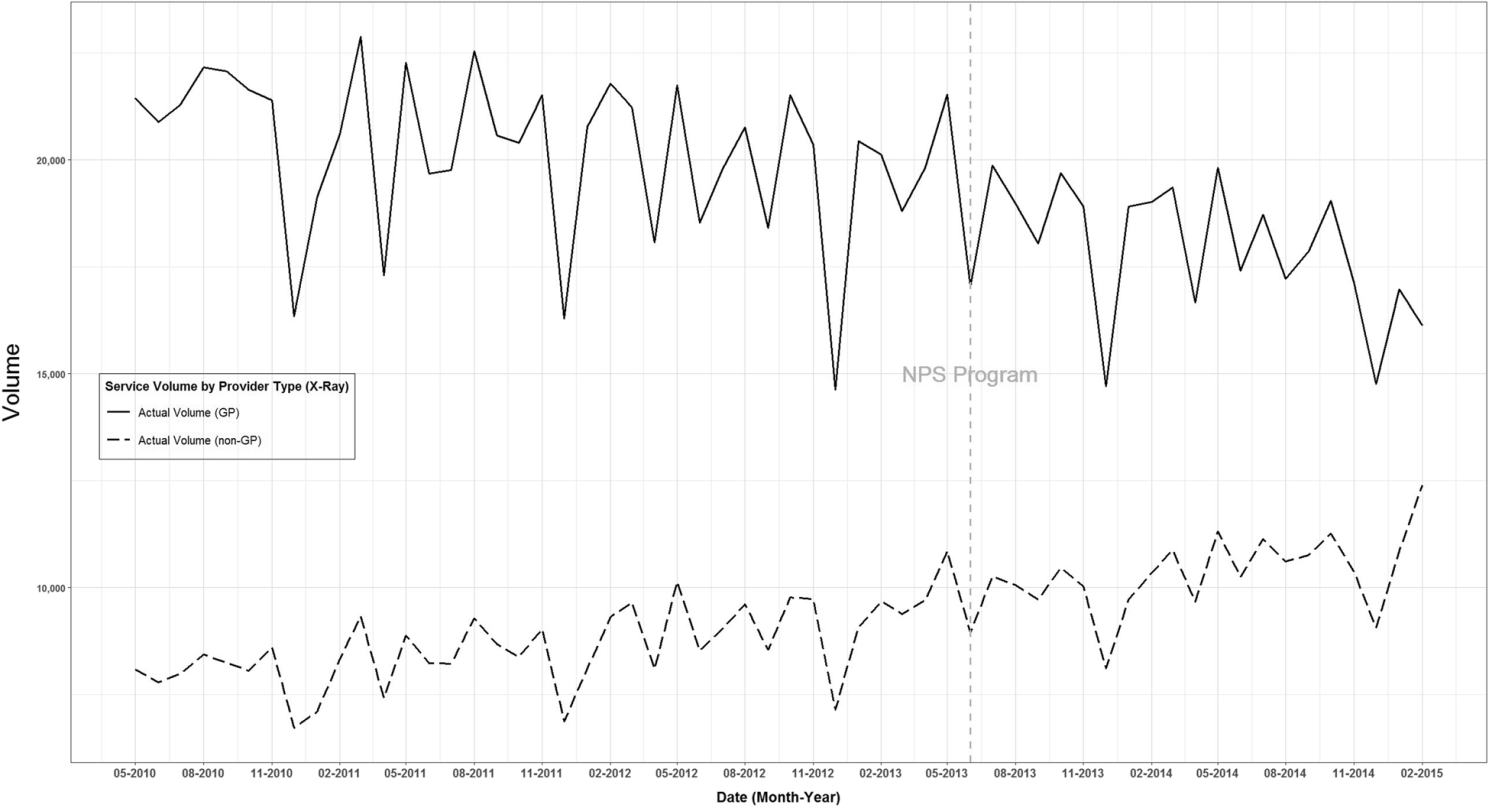
Time series analysis of monthly count of X-ray service volume (item numbers 58106 and 58109), May 2010–February 2015, by provider type for general practitioners and other health professionals

The BHTS method could not be used to analyse X-ray service volume (MBS item numbers 58106 and 58109) due to a lack of statistical stationarity and similarity between the GP and non-GP time series (Figure. 3). Conventional time-series analysis, using an autoregressive moving average model, indicated that the 2013 NPS MedicineWise LBP program did not impact X-ray volume (analysis not shown). There was a decreasing trend in X-ray service volume over time, in contrast to the increasing trend observed in the CT scan time series in Figure. 1.

A summary of results is presented in Table 1.

**Table 1:** Summary of program cost and benefits

Parameter	AUD
Total cost of program implementation	$141,154
Benefit from reduced expenditure on CT scans	$11,575,439
Net Benefit	$11,575,439 - $141,154 = **$11,434,285**
Benefit to cost ratio	$11,575,439 / $141,154 = $**82.01**

### Cost to develop and implement the program

Program costs were estimated to be between AUD$73,734 and AUD$195,960. A cost of AUD$141,154 was used as the base case, based on the best available estimates of costs. Based on disaggregation of the main components’ costs, the program cost AUD$7.05 per GP receiving the intervention. The online decision support tool, MBS feedback report and symptom self-management pad accounted for 41.0%, 40.6% and 4.2% of the program’s cost, respectively. Infrastructure and support services accounted for 14.2% of costs.

### Cost and benefit of the program

The net benefit of the 2013 NPS MedicineWise LBP program to the Australian Government was estimated to be AUD$11.4 million (Table 1). The cost-benefit ratio was calculated by dividing the estimated benefit from reduced MBS expenditure by the cost of the program, giving a ratio of 1:82 (Table 1).

### Cost-effectiveness of the program at reducing CT scans

The incremental cost-effectiveness of the program at preventing CT scans of the lumbosacral region was calculated using ‘no intervention’ (and thus, no costs) as a hypothetical comparator. The 2013 NPS MedicineWise LBP program cost approximately AUD$141,154 to implement. The CT scan service volume in the comparator scenario was defined as the modelled counterfactual estimate without the intervention, presented in Figure. 1. The outcome of CT scans averted was 50,076 for the NPS MedicineWise program and zero for the comparator scenario.

The ICER was $2.82 per lumbosacral CT scan averted. Thus, from the perspective of the Department of Health, the AUD$2.82 spent to avert a lumbosacral CT scan was less than $231 average cost of the CT scan to the MBS, making the program dominant to the comparator of no program.

### Sensitivity analysis

Following a ‘best and worse’ case scenario sensitivity analysis, the cost-benefit ratio for the program’s effectiveness at reducing CT scans ranged from 1:5.57 (maximum estimation of costs and minimum estimation of benefit) to 1:299.07 (minimum estimation of costs and maximum estimation of benefit).

**Fig. 4:**
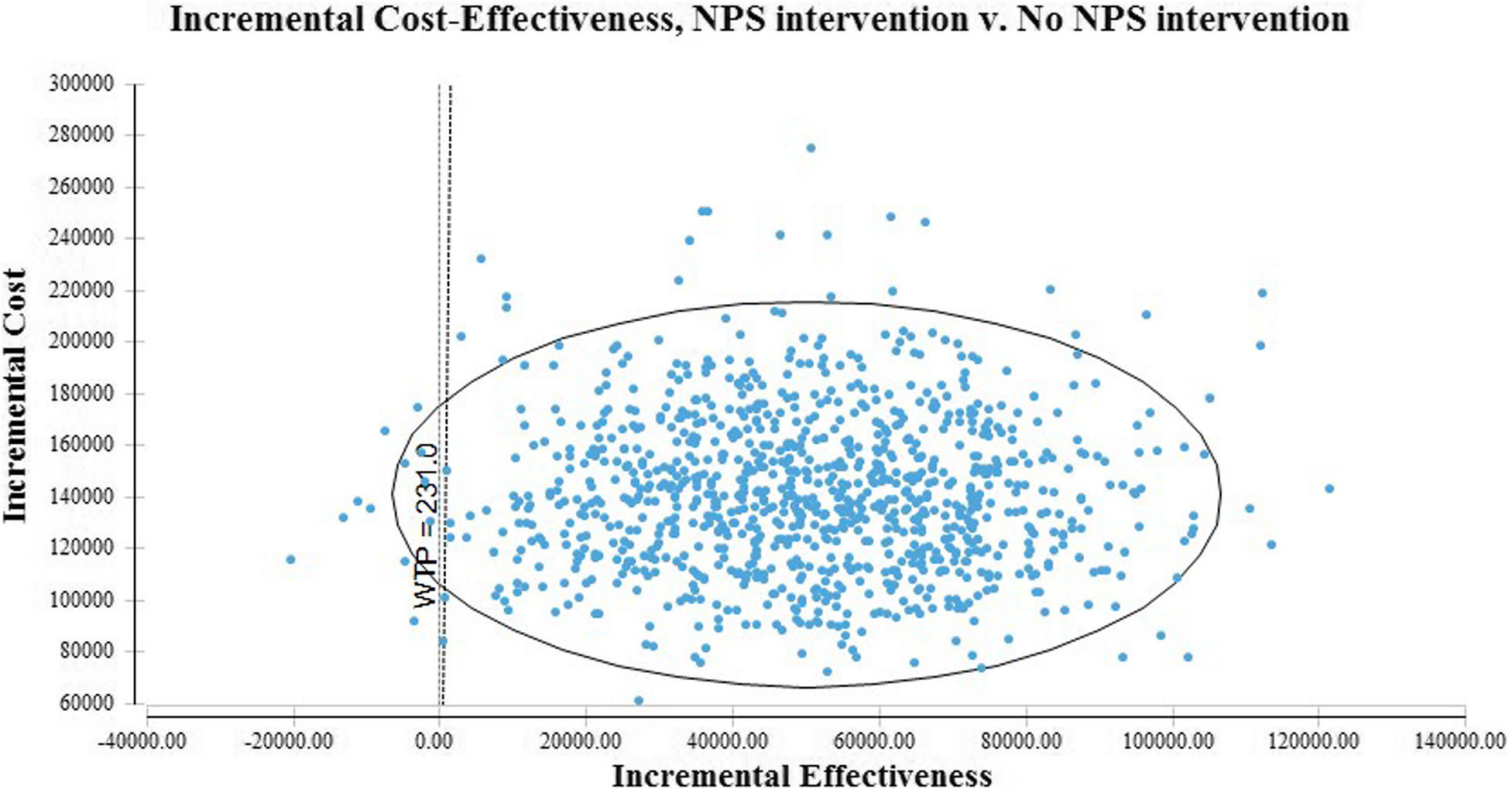
Probabilistic sensitivity analysis plot of incremental cost-effectiveness

Univariate sensitivity analysis of the ICER estimates gave an ICER range of AUD$1.47 to AUD$36.10 per CT scan averted. This range is lower than the AUD$231 willingness to pay threshold, which is the average MBS reimbursement for a lumbosacral CT scan. The ICER was most affected by introduction of the uncertainty in the estimate of the program’s impact. The 2013 NPS MedicineWise LBP program had an ICER less than AUD $231 in 98.3% of iterations in the probabilistic sensitivity analysis (Figure. 4) and was less effective at greater cost in only 1.6% of iterations (Table 2).

**Table 2:** Results from incremental cost-effectiveness plot report

Proportion of iterations from probabilistic sensitivity analysis	Iterations
NPS MedicineWise program more effective, ICER less than AUD$231	98.3% of iterations
NPS MedicineWise program more effective, ICER greater than AUD$231	0.09% of iterations
NPS MedicineWise program inferior – less effective and greater cost	1.6% of iterations

## Discussion

The 2013 NPS MedicineWise LBP program aimed to reduce the number of inappropriate GP referrals for CT scans and X-rays for non-specific acute LBP. This study aimed to quantify the impact of the program on MBS service volume and reimbursement for CT scans and X-rays to evaluate the program’s cost-benefit ratio and cost-effectiveness. The 2013 NPS MedicineWise LBP program was associated with a statistically significant 10.85% relative reduction in the volume of GP-referred CT scans of the lumbosacral region, equating to a cost reduction to the MBS of AUD$11,600,898. No association between the program and the volume of GP-referred X-ray items on the MBS was observed. There was no evidence of a net increase in X-rays associated with the LBP program, indicating that the averted CT scans were not being replaced by additional X-rays. It was not possible to determine if there was a shift in the way X-rays were being used.

This was an observational study so we cannot infer that implementation of the program directly caused the reduction in number of CT scans. However, no other national program or change to practice guidelines were identified over the analysis period. In addition, we used non-GP health professionals as an observational control to account for unidentified events that may have influenced imaging volume, for example access to radiology services. Therefore, the reduction in CT scans of the lower back reimbursed by the MBS was likely due to the 2013 NPS MedicineWise LBP program.

The 2013 NPS MedicineWise LBP program was estimated to cost AUD$141,154. Every dollar spent on the program saved AUD$82 of funding to the MBS for CT scan reimbursements. The program cost AUD$2.82 per lumbosacral CT scan averted compared with the scenario of ‘no program’. A conservative willingness to pay threshold of AUD$231 was used in our sensitivity analysis because it represented the average savings to the MBS from each lumbosacral CT scan averted. The cost saving nature of the program was robust; the program had an ICER of less than AUD $231 in 98.3% of iterations in the probabilistic sensitivity analysis.

Varied results have previously been reported in reviews of interventions intended to improve the management of LBP. [18–20] Jenkins et al. (2015) reported that interventions involving clinical decision support and targeted reminders had the largest impact on reducing imaging for LBP. However, these findings were based on a small number of studies. Interventions involving audit and feedback had variable results while interventions based on practitioner education and guideline dissemination did not reduce imaging for LBP. [19] Mesner et al. (2016) reported no clear association between the type of intervention used and effectiveness at improving the management of non-specific LBP but did conclude that increased frequency and duration of implementation is associated with better outcomes compared with one-off, short-term interventions. [20] Although the heterogeneity of their study was large, Suman et al. (2016) reported that multifaceted implementation strategies were no more effective than minimal, single or no implementation strategies at improving referral behaviour of health professionals. [18]

The 2013 NPS MedicineWise LBP program consisted of interventions based on audit and feedback, decision support and information provision. It was predominately a one-off, short-term intervention that featured audit and feedback (the MBS feedback report) as the widest-reaching intervention. The audit and feedback intervention was similar to one implemented by NPS MedicineWise two years earlier, adding support to the notion that repeated interventions are important to sustain behaviour change. While the 2013 NPS MedicineWise program included interventions with a lower intensity and burden on the GPs involved than other successful Australian programs that aimed to improve the management of LBP, such as the IMPLEMENT trial, [17] it’s strength was that it was implemented nationally. Because the NPS MedicineWise program was implemented and evaluated nationally, no selection bias was introduced through selective recruitment and the evaluation was well powered to detect change. The use of MBS referral data in our study provided our evaluation with a valid and strong data source as it was a national census of GP-referred MBS-funded CT scans and X-rays and was not impacted by self-report bias.

Several limitations should be considered when interpreting the results of our study. First, costs associated with program design and implementation were estimated retrospectively. However, audit and feedback interventions are regularly implemented at NPS MedicineWise for different therapeutic programs so multiple programs were available for our cost estimates. In addition, our sensitivity analysis demonstrated that the program was cost-saving even with variation in the cost estimate. Second, it was not possible to measure how appropriate observed changes in GP referral behaviour were as MBS data do not include reasons for test referral. We were unable to determine whether the reduction in GP referral for CT scans was associated with unintended consequences, such as missed or delayed diagnosis. As a result, we have assumed that the reduction was of unnecessary CT scans, consistent with an economic evaluation of a program promoting the same LBP guidelines [5] as our program. [17] We expect that the promotion of materials raising awareness of guideline recommendations and ‘red flags’ for serious underlying conditions would have improved the detection of patients expected to benefit from diagnostic imaging as well as those expected to have low benefit. In addition to reducing healthcare costs and freeing finite resources, the additional positive consequence of reduced unnecessary CT scans is a reduction in cancer risk associated with the exposure to medical radiation. The evaluation was unable to exclude the possibility of some substitution of imaging by GPs with increased referrals to, and imaging referrals by, medical specialists. Due to these limitations, both downstream benefits from reducing unnecessary radiation burden and downstream costs from potential increased use of imagining referred by medical specialists and any potential reduction in appropriate CT scan use leading to delayed diagnosis, were excluded from the scope of this study. Future research on the sustainability of the impact of this type of intervention would be valuable for decision makers.

## Conclusion

This study demonstrated the effectiveness of a large-scale program at improving clinical practice in line with evidence-based guidelines. The 2013 NPS MedicineWise LBP program was associated with a decrease in CT scans of the lower back referred by GPs. The findings of the economic evaluation suggest that addressing areas of low-value care in the primary care setting with targeted interventions can have a positive financial impact on the health system and reduce patient exposure to clinical interventions that may carry more health risks than benefits.

## Data Availability

The data that support the findings of this study are available from The Australian Government Department of Human Services (DHS) but restrictions apply to the availability of these data, and so are not publicly available. Requests for the data should be made directly to DHS.

## Abbreviations

AUD: Australian Dollars
CT: Computed Tomography
DHS: Department of Human Services
GP: General Practitioner
ICER: Incremental Cost Effectiveness Ratio
MBS: Medicare Benefits Scheme
MRI: Magnetic Resonance Imaging
